# What Are the Peer Interaction Strengths and Difficulties in Children with Developmental Language Disorder? A Systematic Review

**DOI:** 10.3390/ijerph17093140

**Published:** 2020-04-30

**Authors:** Vanessa Lloyd-Esenkaya, Ailsa J. Russell, Michelle C. St Clair

**Affiliations:** Department of Psychology, University of Bath, Bath BA2 7AY, UK; v.e.r.lloyd@bath.ac.uk (V.L.-E.); A.J.Russell@bath.ac.uk (A.J.R.)

**Keywords:** developmental language disorder, peer interaction, social skills, systematic review

## Abstract

The current review gathers together research investigating peer interaction skills in children with Developmental Language Disorder (DLD) to give an overview of the strengths and challenges experienced by these children when interacting with other children. A systematic review was conducted to summarise the literature on peer interaction strengths and difficulties in children with DLD. No restrictions on time-period were made and the selection criteria accounted for many of the diagnostic labels previously used to refer to DLD. Studies included in this review involve English-speaking children of UK primary school age (4–11 years). A systematic search of databases identified 28 papers that met the inclusion criteria. Children with DLD are found to experience many challenges when interacting with peers. Difficulties have been found in studies exploring discourse characteristics such as turn-taking and in behaviours during play, such as access behaviours. Heterogeneity was however notable and peer interaction strengths are found in terms of the children’s abilities to make friends, use verbal and non-verbal behaviour to make joint decisions with peers, and abilities to engage with peers in social pretend play. While it is encouraging to find research exploring many different areas of peer interaction competence in children with DLD, the research is highly disparate and there are many research findings awaiting replication. The current evidence base is unable to comprehensively define the characteristics of peer interactions of children with DLD.

## 1. Introduction

Developmental Language Disorder (DLD) affects approximately 7.6% of 4–5 year olds in the UK [1]. It is diagnosed when children have significantly impaired expressive language and/or receptive language skills in the absence of any hearing or other neurodevelopmental disorder [2]. Children with DLD maintain lower than average language levels throughout their development, and so continue to lag behind their peers throughout childhood and beyond [3,4]. While there is typical development aside from a primary problem with language [5], it is common for children with DLD to show some level of difficulty in areas of attention, motor skills, and social skills [2]. The present study aims to establish the nature of the strengths and difficulties children with DLD display when interacting with peers by systematically reviewing the research in this area. It is difficult to devise strategies to support children with DLD in their social development without a deep insight into possible mechanisms underlying their social difficulties. Establishing the specific nature of peer interaction skills in children with DLD is a critical first step.

Longitudinal studies of large population cohorts have established evidence of peer problems amongst children with DLD [6,7]. Cross-sectional studies similarly find children with DLD to be rated by teachers as having lower social skills, and they are also found to have fewer peer relationships [8], findings which suggest an association between peer problems and longer-term negative consequences such as the inability to maintain friendships.

One characteristic social behaviour often cited as being common in children with DLD is social withdrawal [9,10,11,12]. Studies, again using teacher-rated questionnaires, find children with DLD to have significantly higher levels of social withdrawal compared to children without DLD [13,14]. While the severity of the children’s language difficulties alone do not predict levels of reticence, emotion regulation skills together with language skills can predict reticence in children with DLD [15]. This finding has led researchers to assume children with DLD are fearful of social situations, not just due to having a communication difficulty, but due to difficulties in emotional development [13]. This idea ties in with a previously proposed theory termed The Social Deviance Model [9]. This theory proposes that children with DLD might have inherent difficulties with their socioemotional development, independent of their language difficulties, and it is these socioemotional issues which result in challenges with socialising with other people [9]. The available research into social withdrawal in children with DLD provides a promising, albeit tentative, insight into the underlying reasons for some of the social characteristics observed in children with DLD. However, it is not clear whether social withdrawal is the key behaviour contributing to the peer problems experienced by children with DLD. For example, it is not known whether social withdrawal affects all children with DLD and if not, whether these other children still experience peer problems.

A systematic review and meta-analysis has shown children with low expressive language skills and low receptive language skills have higher levels of behavioural problems, pointing to the possibility that underlying reasons for peer problems in children with DLD might extend beyond simply social withdrawal [16]. Many studies investigating the behaviour of children with disordered language find evidence of externalising problems, which includes angry, oppositional and aggressive behaviour [17,18,19]. More research is necessary to understand whether these externalising behaviours are displayed during social interactions, thus accounting for some of their “peer problems”. It could be that children who lack the expressive language skills necessary for a clear expression of their needs use aggressive behaviour instead of using language. Indeed one study observed behaviour in minimally verbal autistic children and found those who displayed challenging behaviours did so in place of requests, or rejections [20]. Alternatively, disordered receptive language skills might impair the ability to understand social situations and so internal thinking could be relatively immature and involve inappropriate thoughts about other’s intentions, and this could result in an inappropriate response to the situation, such as using physical aggression [19]. It will therefore be a useful endeavour to establish any consistency across research findings about the nature of externalising behaviours and their relation to peer interactions in children with DLD.

Despite evidence that children with language disorder risk developing peer problems, few interventions exist to support their social development. A systematic review conducted in 2012 found only eight studies assessing interventions to support social communication in children with disordered language skills [21]. All of these were exploratory studies, involving samples of less than 20 children, to test the feasibility of interventions, suggesting research in this area is still in its infancy. All eight studies focused on improving the children’s discourse skills, thereby assuming improved discourse skills will lead to enhanced social interaction skills. For example, some focused on improving comprehension skills, in terms of repairing communication breakdowns with their communication partner or monitoring their understanding during conversations [22,23]. In this way, current social skills interventions for DLD build on the premise that their peer interaction difficulties are a direct result of their language difficulties.

To our knowledge, no systematic review of research investigating the social interactions of children with DLD has yet been conducted. In the current study, ten databases are searched using broad search terms with no restrictions on year of publication to capture as many studies as possible exploring this research area. The aim of the current study is to systematically review the findings of studies of peer interaction involving children with DLD in order to establish any consistency as to the specific nature of their strengths and difficulties when socialising with peers. This may enhance our understanding of the potential mechanisms underlying the peer difficulties shown in this population and assist the development of effective, tailored interventions.

## 2. Materials and Methods

The current review includes a systematic search of the literature and narrative synthesis of the research on peer interaction skills in children with DLD.

### 2.1. Search Strategy

Many different terms have been used to define DLD. The current review aimed to capture as many studies of peer interaction characteristics in children with DLD as possible, regardless of how DLD had been defined in the past. To this end the following terms were included as acceptable terms to define language impairment: Developmental Language Disorder (DLD), Specific Language Impairment (SLI), expressive language disorder, mixed expressive-receptive language disorder, previously identified language impairment, language delay, language learning impairment, language disability, language problems, developmental aphasia and developmental dysphasia. These terms were based on a review article [24], and the indexed terms recorded in the included databases. No restrictions were placed on the year of publication, or on the study design.

Searches were conducted from March 2018 to May 2018 using the following databases; PubMed, Embase, Web of Science Core Collection, Web of Science BIOSIS Citation Index and SciELO Citation Index, PsycNET (PsycINFO), ERIC, Proquest International Bibliography of the Social Sciences, Dissertations and Theses A&I, and Ovid (Social Policy and Practice). The search terms included variations of the words “children”, “interaction” and “Developmental Language Disorder”, and the Boolean operators AND and OR were used. Search terms were grouped into three searches which were inserted in the same way in every database (see Appendix A).

### 2.2. Inclusion Criteria

To be included in the review, studies were required to meet the following criteria:

- Children must be 4–11 years old as this is the age at which children attend primary school in the UK. Furthermore, children who were 3 years old or younger were not included because language abilities at this age tend to be too unstable to make an accurate diagnosis of DLD [3].
- The language impairment must be identified as meeting clinical cut-off scores on a standardised language assessment by a researcher or through formal diagnosis by a speech and language practitioner.
- Even if the child has a comorbid condition, such as emotional and/or behavioural difficulties or Attention Deficit Hyperactivity Disorder (ADHD), the child has been selected for the study because language is their primary area of need.
- It must be an empirical study; Intervention studies could be included so long as they included baseline measures of peer interactions.
- The study must be available in English.
- The study must include a measure of peer interactions. The concept of “peer interactions” is difficult to define in concrete terms. For the purpose of this review, studies measuring peer interaction are deemed to be those which measured children talking to each other, children’s use of gestures when engaging with other children, features of children’s play, children’s abilities to resolve situations of conflict with other children, children’s abilities to initiate play or verbal communication with other children or children’s abilities to access the play or communication already taking place between other children.

There are no restrictions on time period or study design in the review, thus giving an outline of the full breadth of research in this area.

### 2.3. Exclusion Criteria

Studies of children who did not speak English, or who had a first language other than English, were not included in the present review. Studies which only included children with language problems who had Autism Spectrum Disorder (ASD), hearing loss, otitis media, or an identified genetic condition known to cause language problems (for example Downs Syndrome, or Fragile X Syndrome), were not included, in line with guidelines for a diagnosis of DLD [2].

### 2.4. Quality Assessment Procedure

The scientific quality of the studies included in this review was assessed using a framework which appraises the quality of both quantitative and qualitative literature [25]. Nine criteria were used to assess quantitative studies and eleven criteria were used to assess qualitative studies (see Appendix B). For each criterion a rating of 1 (low) to 3 (high) was assigned depending on how well the study fulfilled the criterion guidelines. A criterion guide, giving a description of the evidence required to meet a score of 1, 2, or 3 was created by the first author, using the framework provided by Alderfer et al. (2010) [25]. The average of these criteria divided by the number of criteria assessed gave the final quality score.

## 3. Results

**Figure 1:**
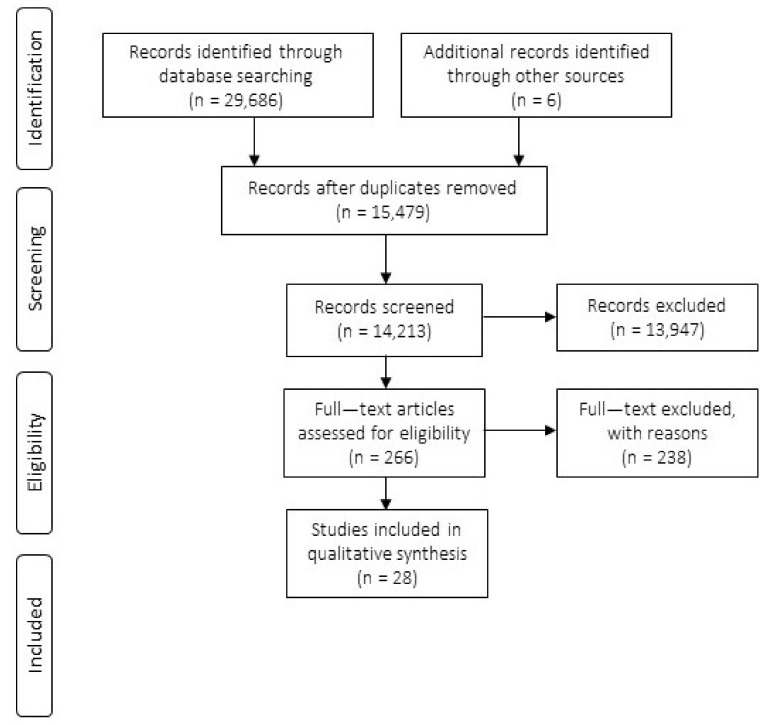
Prisma flowchart to show study selection process [27].

The initial search identified 29,686 records. A further 6 items which were not captured by the initial database searching, but were part of a relevant review [21], were also identified. After duplicates were removed 14,213 unique titles and abstracts were screened for eligibility. Any items which were irrelevant to the subject of peer interactions of children with DLD were excluded at this stage. This resulted in 616 studies being included in the abstract screening stage. Initially, 398 studies were excluded from the review at the abstract screening stage. The main reasons being that children did not meet inclusion criteria for age or native language, or the study was not empirical, or the study did not measure peer interactions, for example mother–child interactions were measured instead. A second-rater screened 10% of these studies (interrater reliability 79.4%). All disagreements were discussed until a mutual understanding was met. Consequently, the primary investigator revisited all items excluded from the initial abstract screening. A decision was made to include a further 48 items which had abstracts providing too little information regarding the subjects or methods to justify an exclusion at this stage. In total, 266 studies were included in the full text screening. Ten percent of the studies included in the full text screening were screened by a second-rater (interrater reliability 91.3%). In total, 28 studies were deemed eligible for inclusion in the systematic review (see Figure 1 and Table 1). Figure 1 has been adapted from the PRISMA flowchart made freely available online [26,27]. See Table 2 for a summary of findings from the 28 included studies.

### 3.1. Overview of Studies

Most of the studies included in this review (60.7%) took place in the United States of America, while others took place in the UK (25.0%), Canada (10.7%) and Australia (3.6%). Some of the included studies come from grey literature (14.3%), specifically PhD theses. While some studies (28.6%) have been published within the last 10 years, a sizeable proportion of studies (35.7%) were conducted before the year 2000, with half of these conducted before 1990.

The studies included in this review involve a total of 856 children with DLD. Twenty-three studies were cross-sectional and five were longitudinal. Of these longitudinal studies three reported attrition rates during data collection at the age range included in this review [7,10,31]. Attrition ranged from 17.4–22.0% and resulted from the researchers being unable to locate the participants or from participants failing to return questionnaires. One study, containing eight children, did not report the gender of the sample [35]. Of the remaining 848 children, 227 are female and 621 are male. None of the children included in this review were reported to have co-occurring conditions. Over half of studies (57.1%) did not identify any co-occurring conditions, while the remaining studies (42.9%) did not report whether children with co-occurring conditions were included. Different age groups are reasonably well represented. The age range of the children with DLD is wider in some studies than others. By looking at each year group separately we find studies include children who are within the age range of 4–5 years (42.9%), 6–7 years (67.9%), 8–9 years (50.0%) and 10–11 years (32.1%). See Table 3 for an overview of the type of school children with DLD were enrolled in across all included studies.

### 3.2. Quality Appraisal

Alderfer et al.’s (2010) quality appraisal framework [25] was used to assess the quality of all the included studies. Studies using quantitative measures were assessed on parameters which included statistical power and appropriate methods. These were felt to be particularly important for the current review of the strengths and difficulties in the domain of peer-based social skills reported by studies of primary school children with DLD. Studies with high statistical power with a high level of internal validity are valuable for this review because this ensures the results of the study are generalisable to other primary school children with DLD.

A second-rater independently appraised the quality of 36% of the papers. Using item-by-item agreement, the two raters were found to agree on 64.4% of quality scores. Inter-rater agreement was low for some of the papers. In particular there was disagreement on the scoring criteria used for categorising papers as low, medium or high quality for the reliable measurement of their variables and whether their statistical power was sufficient. As a result, the two raters engaged in detailed conversations about what criteria would be necessary to achieve the highest quality score on these measures. Previously, for example, there was disagreement over the quality scores given for level of statistical power if the study included fewer than ten children. Following discussions, the raters agreed that medium quality was the maximum score studies including fewer than ten children could achieve for sufficient statistical power. The raters jointly analysed each of the 36% of papers and any disagreements were discussed until 100% agreement was reached on all of the scores. These jointly agreed scores were used for the final quality scores (see Appendix C).

The studies included in this review achieved total quality scores in the range of 1.33 and 3.00 (mean = 2.47, SD = 0.36). In line with previous literature [25] studies with a total quality score at least one standard deviation below the mean (rating < 2.12) were treated as having low scientific quality (*N* = 3, 11%). In this review, studies with a total quality score at least one standard deviation above the mean (rating > 2.82) were deemed to have high scientific quality (*N* = 5, 18%). Low quality papers had small sample sizes and therefore scored low on statistical power and low or medium on external validity. Low quality papers also used their own scoring schemes to measure observed peer interactions and therefore scored medium on the criteria for appropriate methods because while their methods were appropriate, they did not provide enough detail to allow for replication.

High quality papers scored high on criteria for appropriate methods because they used questionnaires such as the Strengths and Difficulties Questionnaire which is easily replicated (SDQ, [53]). Four of the five high quality papers scored high on statistical power and external validity too because their samples included at least 30 children. Those with high scientific quality are highlighted in the text and are useful for interpreting the current evidence base. Those with low scientific quality are retained for this review and are given merit because despite the low generalisability of their findings due to their small samples, their observations provide an important insight into the possible strengths and difficulties children with DLD have when interacting with peers.

### 3.3. Peer-Based Social Domains Measured

We have identified five different skill areas which are investigated in the included studies: overall peer competence, behaviour during play opportunities, discourse characteristics, cooperative behaviour and victimisation. Table 4 provides an overview of the ways in which these skills domains have been explored. It is possible some of these skill areas overlap with respect to the constructs the researchers were aiming to assess. For example, the rationale for investigating discourse characteristics may have been to gain an insight into the children’s play with peers. We categorise the research in this way to allow for greater ease of interpretation. Note that studies are not mutually exclusive; Some studies include measures on more than one relevant peer interaction variable and some studies include measures spanning more than one skill area.

A range of informants were used in different studies measuring overall peer competence including teacher-reports (46.1%), teacher-reports in combination with self-reports (7.7%), teacher-reports in combination with peer-reports (7.7%) parent-reports (15.4%), a combination of teacher and parent reports (15.4%), or direct observation (7.7%). No studies used questionnaire methods in combination with direct observation to measure overall peer competence. All studies measuring behaviour during play opportunities or discourse characteristics used direct observation. Studies which measured cooperative behaviour did so using self-report (33.3%) or direct observation (66.7%). Studies measuring victimisation did so using direct observation (25%), self-report (50%) or teacher-report (25%).

The following narrative synthesis will summarise the available literature on peer interaction strengths and difficulties in primary school children with DLD. It should be noted that 15 (53.6%) of the 28 included studies have not conducted statistical analyses on at least one of their outcome variables which are relevant to peer interaction. This is often due to the small size of the included samples. The results of these studies are therefore largely descriptive which can make it difficult to draw firm conclusions. Nevertheless, the studies summarised in the following narrative synthesis provide a useful foundation on which to build new lines of research.

#### 3.3.1. Overall Peer Competence

Most studies exploring overall peer competence found higher levels of peer problems in children with DLD compared to children without DLD [10,28,31,44,45,48]. Some studies found lower levels of prosocial skills in primary school children with DLD compared to children without DLD [28,31]. There is some discrepancy in the age that peer problems are most pronounced, according to available longitudinal data on primary school children. One recent study finds elevated levels of peer problems at 4 and 5 years, which appear to subside by 7 years [44], yet another study finds elevated peer problems at ages 8 and 10 [45]. Sociometric measures found children with DLD to be less well liked and accepted by their peers compared to children without DLD [38].

There are individual differences in overall peer competence that should be highlighted. One study finds that although some children with DLD have high levels of aggression and some have high levels of withdrawal, other children with DLD have a typical social profile [29]. Similarly, another study found 20% of children with DLD in their sample did not have peer problems [7]. Peer interaction strengths were noted in an earlier study where children with DLD were able to form reciprocal friendships to the same extent as children without DLD [42]. Additionally, some found no differences in the level of prosocial behaviour displayed by children with and without DLD [36]. It seems peer problems are not inevitable for children with DLD because some children have relatively good social skills.

#### 3.3.2. Behaviour during Play Opportunity

Children with DLD were found to interact with their peers on the playground less frequently than children without DLD [11]. There are no conclusive results regarding the type of peer interaction children with DLD are most likely to engage in. In some studies children with DLD are found to engage in non-play more often than play when given the opportunity to interact with peers [51] and high levels of active withdrawal from peers are observed [11]. On the other hand, others find non-play, onlooker behaviour to be rare, with children with DLD showing high levels of interaction with peers on the playground [49]. Furthermore, children with DLD are able to engage in pretend play with peers, with some showing more sophisticated levels of pretend play than children with typical language development [34]. Interestingly, one study found children with DLD mainly engage in social-conversation with peers when they are interacting with peers during free-play, and rough-and-tumble play is rare, suggesting children with DLD do not use more physical forms of play to overcome their language difficulties [11].

Each of the studies measuring access behaviours find children with DLD generally display difficulties accessing play [33,46,47]. Children with DLD use passive social entry patterns [47], they rarely approach peers [33], and they wait longer for an invitation by their peers [46].

#### 3.3.3. Discourse Characteristics

The results from studies measuring discourse characteristics during peer interactions by children with DLD are highly mixed. Studies find children with DLD experience difficulties maintaining conversation with their peers [37]. Children with DLD direct fewer requests to their peers compared to adults [52]. They also ask fewer internal state questions when they are paired with same-age peers compared to when they are paired with younger children [37]. Additionally, children with DLD are more likely than children without DLD to reintroduce topics that have already been introduced, suggesting they find it harder to introduce new topics of conversation [35]. These studies therefore demonstrate that children with DLD experience difficulties during talk with peers. However, a word of caution is needed, because two of the studies [37,52] included fewer than ten children and therefore these results require further replication.

On the other hand, many studies found surprising strengths in the discourse characteristics of children with DLD. They use conversation as a way of seeking information from their partner no less often than children without DLD [49] and are able to talk about rules and plans to engage in pretend play with their peers [43]. Indeed, it seems that play might facilitate discourse in children with DLD. Peers make other-directed turns more frequently during play than between play intervals, and this seems to help children with DLD maintain conversations because their partner can create a shared referent for them to build on and use to make requests [32]. During pretend play, children with DLD are also able to share scripts using non-verbal behaviour [43]. Again, however, these findings come from small sample sizes; Craig and Gallagher’s (1986) [32] findings come from a single case study. These positive findings are therefore in need of replication before they can be trusted.

#### 3.3.4. Cooperative Behaviour

Cooperative behaviour has either been measured in primary school children with DLD using tasks which require children to work together to complete a group activity and their behaviour or discourse is observed, or by presenting children with hypothetical situations and asking how they would behave. Numerous studies find evidence of poor conflict resolution skills in children with DLD [28,30,48]. Unlike typically developing children who ask for clarification from their peers to understand their motives for their actions leading to the conflict event, children with DLD select less sophisticated conflict resolution strategies, such as involving an adult or physical retaliation. It has been suggested that children with DLD have a less nuanced understanding of peer conflict situations than children without DLD because they provide less precise judgements about conflict resolution strategies compared to children without DLD using a visual analogue scale task [30]. There are mixed results regarding the ability of children with DLD to collaborate with their peers. Children with DLD are found to produce fewer validating comments during cooperative tasks than children without DLD [38], and children with DLD who show high levels of withdrawal or high levels of aggression are found to perform poorly on cooperative tasks [29]. Furthermore, children with DLD paired with other DLD children are found to take longer to reach group decisions compared to typically developing dyads [41]. It therefore seems that children with DLD find it difficult to work with their peers to achieve shared goals.

On the other hand, other children with DLD who are scored as having a typical social profile, display fair levels of cooperative skills that are no worse than their typically developing peers [29]. Furthermore, when children with DLD make group decisions they successfully use non-verbal behaviours to do so, in addition to using verbal utterances at the same rate as children without DLD [41]. This therefore suggests poor cooperation skills affect only some children with DLD and, as with other areas of social competence in children with DLD, there are individual differences here.

#### 3.3.5. Victimisation

Only four studies included measures of victimisation [11,29,31,50]. There is evidence showing children with DLD have an elevated risk of being victimised by their peers at age 7–8 [50] and at age 11 [31]. The study by Fujiki et al. (2001) [11], however, finds no difference in levels of victimisation between children with and without DLD at age 6–10. The study by Conti-Ramsden and Botting (2004) [31] includes a large sample size of two-hundred children with DLD. These more reliable findings indicate there is an increased risk of victimisation among children with DLD. The discrepancy in the present findings on victimisation in children with DLD may result from differences in measurement techniques, with self-report measures being using in some studies [31,50] and direct observation being used in others [11]. The peers of children with DLD are possibly less likely to engage in bullying while they are being observed by an adult, and therefore self-report measures might more accurately portray levels of victimisation of children with DLD.

## 4. Discussion

This systematic review with narrative synthesis sought to refine our understanding of the nature of peer interaction strengths and difficulties shown by children with DLD. Of the studies reviewed, the heterogeneity of skills domains studied, and range of measures used means synthesising findings across a disparate literature is complex. The quality appraisal found most studies (71%) had medium scientific quality. Few studies (18%) had high scientific quality. In this review, the studies with the highest scientific quality are mostly those using questionnaires which measure overall peer competence, because these methods are described in enough detail to be replicated. However, findings from these types of studies provide minimal details into the specific nature of the peer interaction strengths and difficulties experienced by children with DLD. This review has revealed a dearth of research with high scientific quality investigating specific aspects of peer interaction in children with DLD. Nevertheless, those lower quality studies measuring distinct peer interaction characteristics are valuable in providing a basis from which to conduct further focused research on the social development of children with DLD. The available evidence points to the idea that primary school children with DLD struggle to access play and have poor conflict resolution skills. The outcomes of research into the play behaviour and discourse characteristics of these children are less clear but they raise some interesting questions and have the potential to form the foundation for a fascinating new area of research.

Children with DLD are a heterogenous group in terms of the nature and severity of their language difficulties [54]. It is clear from the available evidence that children with DLD are also heterogeneous in their social skill levels. While this review has found studies are consistent in reporting elevated levels of peer problems in primary school children with DLD [10,28,31,44,45,48], not all children with DLD seem to experience difficulties interacting with peers or making friends [7,29,42]. The reasons some children with DLD demonstrate more competent peer interaction skills than others are unclear. In the study by Brinton et al. (2000) [29] there was not a direct relationship between language disorder severity and language profile, suggesting there are additional factors which contribute to overall social skills when children have DLD. Indeed, there are a wide range of variables which might influence the children’s social skills. For example, their level of pragmatic skill, such as turn-taking ability during conversation, and the characteristics of the children in their peer group, such as whether the other children in their class also have language difficulties. The study by Mok et al. (2014) [7] finds evidence indicating children’s level of prosocial behaviour and emotional symptoms influences their competence socialising with peers. Future research should further unpick the underlying factors protecting some children with DLD from experiencing peer interaction difficulties. If the underlying factors are malleable, it might be possible to develop targeted interventions to support children with DLD, who are experiencing peer problems, in developing better social skills.

In the current literature exploring the peer interactions of primary school children, females with DLD are underrepresented. A higher proportion of boys than girls are clinically diagnosed with DLD [55]. However, an epidemiological study has found the prevalence and severity of language disorder to be very similar across the sexes, with a sex ratio (male:female) of 1.22:1 [1]. The large discrepancy in numbers of girls and boys included in current studies cannot therefore be justified by prevalence rates across sexes. Caution should therefore be taken when generalising current findings relating to the peer interaction skills of primary school children with DLD to girls because the available evidence is more representative of boys than girls.

Almost half of the studies included in the present review made no mention of whether the children in their sample experienced any co-occurring conditions. The studies included in this review were of children who had been selected for the research due to their language difficulties. However, DLD is a highly heterogenous condition [54] with many children having comorbid diagnoses with attention deficit hyperactivity disorder (ADHD) [56] and emotional behavioural difficulties (EBDs) [57]. Research has shown children with ADHD and EBDs are at risk of experiencing social skill difficulties [58,59]. It is therefore important to take comorbid conditions into account when exploring peer interaction in children with DLD. Going forward, it will be useful for future researchers to acknowledge this comorbidity and include more detail about the children represented in their samples.

The studies reviewed here have investigated the peer interaction strengths and difficulties experienced by children with DLD, but there has been more of a focus on difficulties. One reason we currently have little knowledge of the peer interaction strengths experienced by children with DLD may be that the focus of the available studies has not been on the children’s own perceptions of their peer interactions or friendships. While two of the included studies use self-reports to investigate victimisation, the questionnaires employed do not offer children the opportunity to comment on positive features of social interactions [31,50]. One of the included studies used sociometric ratings to measure friendships in children with DLD [38]. The children with DLD and their peers were asked to list their three best friends which gave a measure of the number of reciprocal friendships the children have. It is encouraging to see child-centric methods employed. However, the current findings offer little insight into the perception children with DLD have of their relationships with their peers. Future research could benefit from the use of self-reports that allow children with DLD to report their strengths with regards to peer interactions. One way to do this is to employ qualitative methods. This will come with unique challenges, given the difficulties children with DLD will have in engaging with a verbal discussion. However, arts-based qualitative methods, such as Photovoice may prove feasible for this type of investigation [60]. Furthermore, guidelines on conducting qualitative research with those who have low language abilities, such as those with aphasia, can guide future researchers in this area [61,62]. A more rounded understanding of the peer interaction skills of children with DLD will better enable teachers and Speech and Language Therapists to build on the children’s pre-existing strengths to support their social development.

It can be seen from this review that the peer interactions of children with DLD have been studied both at a macro and micro level and a wide range of different skills domains have been explored. Studies which look at the social interactions of children with DLD in close detail, such as DeKroon et al.’s (2002) [34] study of pretend play and Grove et al.’s (1993) [41] study of joint decision-making, provide exciting avenues of new research because they lay the groundworks from which to base future research projects. Overall, however, research investigating the peer interactions of children with DLD is sparse. Much more work needs to be done to uncover the underlying reasons for the peer problems so far observed in children with DLD [10,28,31,44,45,48].

The most reasonable next step for research in this area might be to combine different skills domains. Play behaviour among children with DLD, for example, remains to be fully explored. While there is some evidence showing children with DLD frequently engage in non-play during playtime [51] and show high levels of social withdrawal [11], the underlying reasons for such withdrawal behaviour are yet unknown and this limits our understanding of the underlying reasons for peer problems in children with DLD. It could be that children with DLD withdraw from social situations because they are unsure how to access peer play [33,46,47]. Studies linking access behaviour to questions regarding social withdrawal have not yet been conducted in primary school children with DLD. Overall, current literature does not provide a coherent picture of the peer interaction skills of children with DLD. Many studies, particularly older studies using observational techniques, investigate very specific aspects of these children’s peer interaction skills. There is little unity between these studies and a lack of replication of findings because each pose different research questions. In contrast, multiple studies from the past two decades investigate peer interaction skills more broadly and present replicable findings because they use standardised questionnaires. However, a limitation of studies using these questionnaire measures is that they provide minimal detail into underlying reasons for the children’s peer problems. Moving forward, research in this area should now investigate the relationships between multiple skills domains to answer specific research questions using replicable research methods. This has started to happen in studies exploring conflict resolution [28,38,48] or victimisation [11,29,50] in combination with overall peer interaction skills. Future research could bridge other skills domains. For example, the same study could measure access behaviour in combination with discourse characteristics, overall peer competence and victimisation.

Another approach to understanding the underlying reasons for peer problems in children with DLD might be to build on methods used in previous studies which explore discourse in children with DLD [32,35,37,39,43,49,52] to find out more about the peer interactions taking place when children with DLD are engaged in play. Studies find children with DLD are able to engage in social pretend play [34] and are able to share referents with their peers [32], which begs the question of why children with DLD are being rated by their teachers and parents as having peer problems [10,28,31,44,45,48]. No studies investigating the discourse of children with DLD have been conducted within the last 10 years. With new technologies, such as the LENA system [63], it is now possible to record the speech of children with DLD in a non-intrusive way while they are engaged in play in naturalistic settings, such as the school playground. These technologies could provide new opportunities to further explore the strengths and difficulties children with DLD experience while they play with their peers.

One area where children with DLD may need support in order to develop good social skills is understanding and managing situations of peer conflict. Studies conducted so far find children with DLD have worse conflict resolution skills than children without DLD [28,30,48]. The findings from the studies included in this review show children with DLD more frequently select conflict management styles which are considered “low-level” including no response, physical retaliation, and submission [28,48]. These strategies place less reliance on verbal skills than so-called higher-level responses, such as asking for clarification. Some have suggested children with DLD rely more heavily on non-verbal strategies to overcome conflict situations as a coping strategy to avoid negotiation [48]. Therefore, poor conflict resolution skills in children with DLD could be a direct result of their low language abilities. However, it is unclear whether the difficulties children with DLD have in managing conflicts result entirely from their expressive and receptive language difficulties or whether they also have a difficulty understanding social situations involving conflicts. Campbell and Skarakis-Doyle (2011) have suggested children with DLD have a less nuanced understanding of conflict resolution strategies and goals than children without DLD [30]. It will be important for future research to address this in order to develop appropriate interventions to support children with DLD. If children with DLD lack a complete understanding of social situations involving conflicts, it will be important for clinicians to address their social awareness and not merely their language skills.

There is evidence to suggest that children with DLD have an elevated risk of being victimised by their peers [31,50]. If this is the case, it will be important for future studies to explore the reasons children with DLD are victimised. One study found poor expressive language skills consistently correlated with victimisation scores, which suggests the skill with which children can communicate their ideas to their peers might influence their risk of being bullied [31]. Another possible risk factor, as shown by a study of victimisation in older children with DLD, is competence in understanding one’s own basic emotions [64]. Possibly, the ability to understand one’s own emotions enables one to more competently mask their negative feelings from a bullying peer, thus preventing the bully from gaining a sense of power, which reduces the risk of further victimisation [64,65]. While a small number of studies investigated the conflict resolution skills of primary school children with DLD, no studies have yet looked at victimisation in tandem with conflict resolution skills. This may therefore open another promising new area of research. It is possible that children with DLD with more advanced conflict resolution abilities are less likely to be victimised compared to other children with DLD. Research shows children who are bullied during childhood have an elevated risk of developing psychiatric problems, such as generalised anxiety and panic disorder, in later life [66]. Indeed, new research shows adolescents with a history of DLD who have experienced bullying have an increased risk of developing internalising symptoms [67]. Protecting children from bullying is therefore important. By understanding why language difficulties increase the risk a child will be victimised, it might be possible to develop appropriate strategies which schools could use to minimise bullying.

While conducting this systematic review, there were certain papers which could not be included due to restraints on age of participating children. Only papers assessing the social interaction skills of children aged 4–11 could be included. Some papers assessed the social interaction skills of children older than, but also including, this age range. If a separate analysis was not provided for children above the age of 11, these papers could not be included, thus certain papers with potentially interesting findings were not included in this review. While a review of literature relating to social development in adolescents with DLD exists [68], to our knowledge, no review has yet focused specifically on the primary school years. It was therefore essential to place restrictions on the inclusionary criteria in this way in order to review what is currently known about the peer interaction skills of children with DLD between the ages of 4 and 11.

It is difficult to conduct a comprehensive review relating to DLD, since many different terms have previously been used to describe the condition [69]. The inclusionary criteria relating to the term DLD was kept broad, to try to include as many variations of the term as possible. Papers could be included if disordered language was identified either through formal diagnosis by a speech and language practitioner, or through language assessment by a researcher. Papers could be included if the sample of children fulfilled all exclusionary criteria and were given a diagnosis of a wide range of possible terms (detailed in our methods section), to reflect historic changes in terminology [69]. Despite these efforts, it is possible that certain papers investigating the social interaction skills of children who would now be considered to have DLD were missed.

## 5. Conclusions

This systematic review has shown that research exploring the peer interactions of children with DLD does exist, but the available literature is disparate in terms of the skills domains being explored. Studies using questionnaire methods generally find children with DLD have a higher prevalence of peer problems than children without DLD. Studies using direct observation, such as those measuring children’s behaviour on the playground or discourse during peer interactions in the lab, provide tentative clues to the underlying reasons for these peer problems, although the results from these studies are highly varied and the relationship between DLD and social competence with peers appears to be highly complex. There is a need for the replication of the findings from these observational studies which tend to use small sample sizes. Future studies could take a more holistic approach by linking together different skills domains within the broad construct of peer interactions.

## Figures and Tables

**Table 1:** Table showing reasons full-text items were excluded from the qualitative synthesis.

Reason Excluded	Total Items Excluded
Did not measure peer interactions	63
Children did not meet criteria for having DLD in the absence of other diagnoses known to impact on language skills	24
Children were not native-English speakers	55
Children were not within the age range 4–11 years	72
Not an empirical study	9
Same sample was described in a later study	14
Search had to be terminated because the item was a thesis and the university holding the thesis was unable to send or lend the item	1

**Table 2:** Summary of Findings.

Study	Number of Children with DLD	Age Range of Children with DLD	Educational Setting of Children with DLD	Primary Purpose of Study	Study Design	Relevant Skills Domains Measured	Relevant Data Collection Measures	Key Findings
Bakopoulou and Dockrell (2016) [28]	42	6–11 years	Mainstream school (29N), specialist language unit attached to mainstream school (13N)	To investigate social cognitive skills in relation to socio-emotional functioning in children with DLD.	Cross-sectional between subjects. Comparison against chronologically age-matched (42N) and language age-matched (42N) TD children.	Overall peer competence: Peer problems, Prosocial behaviour. Cooperative behaviour: Conflict resolution knowledge.	Strengths and difficulties questionnaire (SDQ; (Goodman, 1997): Peer problems, Prosocial subscales. Teacher-report. Observation during “conflict resolution abilities” task, devised by the investigators. Investigators devised their own coding scheme.	**Strengths:** No relevant strengths. **Difficulties:** Compared to age-matched and language-matched TD peers, children with DLD had higher peer problems (*p* < 0.001, *ηp²* = 0.24 ), lower prosocial behaviour (*p* < 0.001, *ηp²* = 0.36), lower conflict resolution task performance (*p* < 0.001, *η² = 0*.22).
Brinton, Fujiki, Montague and Hanton (2000) [29]	6	6–7 years	Mainstream school	To see how individual social profiles influence cooperation skills in children with DLD.	Multiple case study. Triadic interactions with chronological age-matched TD children (48N).	Overall peer competence: Level of social withdrawal, Prosocial behaviour, Social competence with peers. Victimisation: Type of victimisation. Cooperative behaviour: Joint decision making on cooperative tasks.	Teacher Behaviour Rating Scale (TBRS, Hart and Robinson, 1996): Withdrawn, sociable, anxious/distractible, impulsive, hostile/aggressive proactive, hostile/aggressive reactive, victimisation behaviour profiles. Observation during 4 cooperative tasks. Investigators devised their own coding scheme.	**Strengths:** One child with DLD had a typical social profile. * Difficulties: Children with DLD showed little cooperation with many (N = 4) either preferring to work independently or being ignored by their peers. The social profiles for some children with DLD included aggression (N = 1), social withdrawal (N = 1), or aggression and withdrawal (N = 2). The social profile for 1 child included relational and overt victimisation. *
Campbell and Skarakis-Doyle (2011) [30]	6	9–11 years	Not stated	To assess peer conflict resolution knowledge in children with DLD.	Cross-sectional between subjects. Comparison against same age TD children (26N).	Cooperative behaviour: Conflict resolution knowledge.	Experimental peer conflict resolution knowledge task using investigator’s devised visual analogue scale (VAS).	**Strengths:** Children with DLD (N = 6) could accurately identify social scenarios which involve conflict. * Difficulties: Children with DLD assigned ratings to express their conflict resolution strategy preferences to one of three VAS anchors more often (60%) than TD children (36%). Children with DLD express global rather than nuanced preferences. *
Conti-Ramsden and Botting (2004) [31]	200	8–11 years	Mainstream school (55%), specialist language school (3%), language unit attached to mainstream school (25%), other specialist placement (17%)	To investigate the social and behavioural development of children with DLD.	Longitudinal. Comparison with norm data set for victimisation assessment only.	Overall peer competence: Level of social withdrawal. Overall peer competence: Peer problems, Prosocial behaviour. Victimisation: Frequency of victimisation.	Harter Perceived Competence Scale (Harter & Pike, 1984): Peer Competence subscale. Teacher-report. SDQ (Goodman, 1997): Peer problems, Prosocial subscales. Self-report. “My Life in School” Questionnaire (MLIS; Sharp, Aurora, Smith, & Whitney, 1994): Victimisation subscale. Self-report.	**Strengths:** Many children with DLD (60%) did not have peer problems and many (56%) had typical levels of prosocial behaviour. * Difficulties: Some children with DLD (40%) had peer problems at age 10–11. Some children with DLD (44%) had few prosocial behaviours. * Children with DLD had a higher risk of being bullied at school than TD children (*p* < 0.005). Some children with DLD (35%) aged 10–11 experienced at least 3 different types of peer victimisation at least once a week.
Craig and Gallagher (1986) [32]	1	4 years	Not stated	To investigate the relationship between frequency of related responding to comments and interactive play.	Case study. Dyadic interactions with same age (2N) and 2-year old (2N) TD children.	Discourse characteristics: Comments, Other-directed/self-directed utterances, Responses, Requests for clarification. Behaviour during play opportunities: Type of play.	Observation during 20-min play sessions. Investigators devised their own coding scheme.	**Strengths:** Child with DLD made more related responses when engaged in play compared to when engaged in non-play during peer interactions. * Difficulties: Child with DLD showed an inconsistent ability to do related-responding during peer interactions. *
Craig and Washington (1993) [33]	5	7–8 years	Mainstream school (5N)	To compare the verbal and non-verbal behaviours used by children with and without DLD to access established interactions.	Multiple case study. Triadic interactions with age-matched (4N) and language-matched (4N) TD children.	Behaviour during play opportunities: Access behaviours. Verbal and non-verbal task-related behaviours. Task-unrelated behaviours. Discourse characteristics: Personal identifications, remarks, responses, challenges, comments.	Observation during 20-min play sessions. Investigators devised their own coding scheme.	**Strengths:** Some children with DLD (N = 2) were able to access established peer interactions. All children approached their peers at some point and responded to their peers in a task-related way at the start of the session. * Difficulties: Some children with DLD (N = 3) were unable to access established peer interactions. These children ignored their peers when invited to turn-take. These children did not repeat attempts to access after an initial attempt failed, instead engaging in observation of peers of solitary play. *
DeKroon, Kyte and Johnson (2002) [34]	3	4–6 years	Not stated	To investigate the social pretend play and discourse behaviour of children with DLD when playing with peers.	Multiple case study. Dyadic interactions with DLD and same age (4N) TD children.	Behaviour during play opportunities: Proportion of social pretend play, mean length of social pretend play, number of social pretend play theme categories. Discourse characteristics: Mean number of conversational turns, proportion of other-directed turns, proportion of maintaining turns.	Observation during 20-min play sessions. Investigators devised their own coding scheme.	**Strengths:** All children with DLD (N = 3) engaged in social pretend play with TD and DLD peers. Children with DLD showed a higher proportion of successful conversational turns during social pretend play compared to non-social pretend play episodes. * Difficulties: Turn-taking was less frequent in DLD-dyads than in DLD-TD dyads and turn-taking in DLD-TD dyads was less frequent than in TD-dyads.*
Edmonds and Haynes (1988) [35]	8	5–7 years	Mainstream school	To investigate the discourse of children with DLD during interactions with normal language peers.	Multiple case study. Dyadic interactions with DLD and same age (8N) TD children.	Discourse characteristics: Topic maintenance/manipulation: Topic maintenance, topic change, topic shading, back-channel responses. Overall peer competence: Level of Assertiveness/withdrawal	Observation during 15–20 min interaction sessions. Investigators devised their own coding scheme. Walker Problem Behavior Identification Checklist (WPBIC, Walker, 1983): Withdrawal subscale. Teacher-report	**Strengths:** No difference in proportion of topic maintenance, topic shading, back channel responses, or topics introduced between DLD and TD children. No difference between DLD and TD children in level of assertiveness/withdrawal. **Difficulties:** Children with DLD made a significantly higher proportion of topic-reintroductions than TD children (*p* < 0.05).
Farmer (2000) [36]	16	10–11 years	Specialist language school (8N), specialist language unit attached to mainstream school (8N)	To investigate social cognition in relation to school placement in children with DLD.	Cross-sectional between subjects. Comparison against chronologically age-matched (8N) and language age-matched (8N) TD children.	Overall peer competence: Peer problems, Prosocial behaviour.	SDQ (Goodman, 1997): Peer problems, Prosocial subscale. Teacher report.	**Strengths:** No significant difference in level of prosocial behaviour between DLD and TD children. No significant difference in level of peer problems between children with DLD attending language units compared to chronologically age-matched TD children. **Difficulties:** Significantly higher peer problem scores in children with DLD attending specialist schools compared to age-matched TD children (*p* value not stated).
Fey, Leonard and Wilcox (1981) [37]	6	4–6 years	Not stated	To investigate the discourse of children with DLD during interactions with normal language peers.	Multiple case study. Dyadic interactions with DLD and chronologically age-matched (6N) TD children, and DLD and language age-matched (6N) TD children.	Discourse characteristics: Internal state questions, External world questions, Imperatives, Attentional utterances, Self-repetitions, Back-channel responses.	Observation during 20–30 min play sessions. Investigators devised their own coding scheme.	**Strengths:** Significantly longer mean pre-verb sentence length (more complex speech) by DLD children during interactions with chronologically age-matched peers compared to interactions with younger, language age-matched TD (*p* < 0.016). **Difficulties:** Significantly more internal state questions directed by DLD children to language age-matched TD children than to chronologically age-matched TD children (*p* < 0.031).
Fujiki et al (2013) [38]	4	6–9 years	Mainstream school (4N)	To find out whether a social communication intervention for children with DLD would increase the production of validating comments during peer play.	Intervention using multiple case studies. Triadic interactions with DLD and chronologically age-matched (2N) TD children.	Cooperative behaviour: Validating comments during cooperative learning tasks. Overall peer competence: Sociometric ratings of peer liking, Ability to form reciprocal friendships, Prosocial behaviour.	Observation during three 20-min cooperative learning tasks. Investigators devised their own coding scheme. Sociometric measures of peer acceptance and friendship using Hart, Ladd and Burleson (1990) procedure. Peer-report. Teacher Behaviour Rating Scale (TBRS, Hart and Robinson, 1996): Prosocial, Impulse control/likability subscales. Teacher-report.	**Strengths:** The level of peer acceptance for one child with DLD was equal to the normative mean at baseline. One child with DLD had reciprocal friendships. * Difficulties: Children with DLD (N = 4) produced fewer than 5 validating comments on cooperative tasks. Some children with DLD (3N) had no reciprocal friendships. These children had lower peer acceptance scores (2.14) than normative mean (2.58). All DLD children (4N) had lower likeability scores (1.00) than normative mean (1.79) at baseline and lower prosocial scores (0.90) than normative mean (1.71). *
Fujiki and Brinton (1991) [39]	1	9 years	Mainstream school	To describe the conversational responsiveness and assertiveness of a child with DLD during interactions with different conversation partners.	Case study. Dyadic interactions with child with DLD and adult (1N), language-age matched TD child (1N), and chronological-age matched TD child (1N).	Discourse characteristics: Amount of talk, Type of content in discourse, Topic maintenance/manipulation.	Observation during 30-min interaction with chronological-age matched TD child. Investigators devised their own coding scheme.	**Strengths:** Child with DLD produced more utterances and higher proportion of assertions than TD child. Child with DLD makes equal number of turn initiations as TD child. * Difficulties: DLD child responsible for high proportion of noncollaborative utterances because frequently makes non partner-directed utterances. *
Fujiki, Brinton, Isaacson and Summers (2001) [11]	8	6–10 years	Mainstream school (8N)	To find out how children with DLD behave socially on the playground.	Cross-sectional between subjects. Comparison with chronologically age-matched TD children (8N).	Behaviour during play opportunities: Type of interaction/play behaviour. Victimisation: Frequency of victimisation.	Observation during 45 min play sessions. Investigators devised their own coding scheme.	**Strengths:** One child with DLD spent as much time engaged in social conversation and rough-and-tumble play as TD children. No difference in level of victimisation between children with and without DLD. * Difficulties: Children with DLD (N = 8) spent less time interacting with peers (*p* = 0.0117) and more time withdrawing socially (*p* = 0.0132) than TD children.
Gibson, Hussain, Holsgrove, Adams and Green (2011) [40]	42	5–11 years	Mainstream school (42N)	To assess the reliability and validity of a new standardised method to observe playground behaviour.	Cross-sectional between subjects. Comparison with age-matched TD children (44N), children with externalising pathologies (44N), internalising pathologies (19N) and Autism Spectrum Disorder (39N).	Behaviour during play opportunities: Prosocial behaviour, Conflict, Care-giving behaviour, Atypical behaviour.	Observation for 10 min during natural playground play sessions Investigators devised their own coding scheme called the Manchester Inventory for Playground Behaviour (MIPO).	**Strengths:** Children with DLD (N = 42) had higher levels of observed prosocial and care-giving behaviours than TD children. * Difficulties: Children with DLD (N = 42) had higher levels of observed atypical behaviours than TD children. *
Grove, Conti-Ramsden and Donlan (1993) [41]	15	6–7 years	Language unit attached to mainstream school (15N)	To see how children with DLD make decisions in conversational contexts.	Cross-sectional between subjects. Dyadic interactions with other DLD children, chronologically age-matched (15N) and language age-matched (6N) TD children.	Cooperative behaviour: Joint decision making on cooperative task, including length of time to make a decision, communication to make a decision, number of winning moves, number of conflict moves and types of decisions.	Observation during cooperative task. Investigators devised their own coding scheme.	**Strengths:** No significant difference between number of verbal winning moves made by children with DLD compared to age-matched controls. Children with DLD made significantly more non-verbal winning moves than age matched controls (*p* < 0.05). **Difficulties:** Dyads with DLD took significantly longer to reach decisions than age-matched controls (*p* < 0.001).
Guralnick, Gottman and Hammond (1996) [42]	30	4–5 years	Not stated	To find out how the social setting affects friendship formation in children with DLD.	Cross-sectional within subjects. Compare interactions of children with DLD with peers in playgroups containing other DLD children and in playgroups containing mainly age-matched TD children.	Behaviour during play opportunities: Social participation, Level of cognitive play. Overall peer competence: Ability to form unilateral and reciprocal friendships.	Observation during sixty-minute play sessions, recorded from ten 2.5h play groups. Investigators devised their own coding schemes, including the Individual Social Behavior Scale (ISBS).	**Strengths:** No difference in the frequency with which children with DLD form reciprocal friendships compared to TD children. No significant difference in the frequency with which children with DLD form reciprocal friendships in specialist compared to mainstream settings. **Difficulties:** Children with DLD who did not form reciprocal friendships observed peers significantly more often (*p* < 0.05) and sought the attention of their peers significantly more frequently (*p* < 0.001) than children with DLD who did form reciprocal friendships.
Lederer (1996) [43]	6	5–6 years	Specialist language school (6N)	To investigate the collaborative pretend play language used by children with DLD during peer play.	Cross-sectional between subjects. Dyadic interactions between children with DLD. Compared to dyadic interactions of language-age matched (3N) and chronological-age matched (3N) TD children.	Discourse characteristics: Type of discourse, including metacommunicative or non-metacommunicative, Content in discourse, Function of discourse, and Form.	Observation during three 20-min play sessions. Investigators devised their own coding scheme.	**Strengths:** Children with DLD demonstrated skill in using pretence during play with other children with DLD. * Difficulties: Children with DLD produced non-metacommunications significantly less frequently than children with typical language development (*p* < 0.01).
Levickis et al (2017) [44]	122	4–7 years	Not stated	To compare socio-emotional and behavioural development across time in children with and without DLD.	Longitudinal. Comparison with TD children included in the longitudinal sample, at every time point.	Overall peer competence: Peer problems, Prosocial behaviour.	SDQ (Goodman, 1997): Peer problems, Prosocial subscales. Parent-report.	**Strengths:** No difference between children with and without DLD in peer problem scores at age 7, or in prosocial scores at 5 and 7, after adjusting statistical models for potential confounders. **Difficulties:** Peer problem scores significantly higher in children with DLD than children without at age 4 (*p* < 0.001) and 5 (*p* = 0.01), after adjusting statistical models for potential confounders. Prosocial scores significantly lower in children with DLD than children without at age 4 (*p* < 0.001).
Lindsay and Dockrell (2012) [45]	69	8–10 years	Not stated	To find out behavioural, emotional and social difficulties, and self-concepts change over time in young people with DLD.	Longitudinal. Comparison with normative sample data at every time point.	Overall peer competence: Peer problems, Prosocial behaviour.	SDQ (Goodman, 1997): Peer problems, Prosocial subscales. Teacher-report.	**Strengths:** No relevant strengths. **Difficulties:** Peer problem scores higher and prosocial scores lower in children with DLD than children than population average at age 8 and 10. * Prosocial difficulties increased between 8 and 10 years in children with DLD (*p* = 0.021).
Liiva and Cleave (2005) [46]	10	6–8 years	Mainstream school	To investigate the ability of children with DLD to access and participate in an ongoing peer interaction.	Cross-sectional between subjects. Triadic interactions with same age TD children (13N).	Behaviour during play opportunities: Type of play behaviour, Behaviour and discourse during play relating to peer access, and whether access was successfully achieved.	Observation during 10-min play sessions. Investigators devised their own coding scheme.	**Strengths:** Six children with DLD did access play with peers. **Difficulties:** Four children with DLD did not achieve access to play with peers. Children with DLD took longer than TD peers to achieve access play (*p* = 0.024). Children with DLD engaged in more onlooker behaviour (*p* = 0.011), less group-play (*p* = 0.000) and more individual play (*p* = 0.005).
Margolis (2001) [47]	19	5–9 years	Mainstream school	To compare the social entry patterns of children with DLD to children with Autistic Spectrum Disorder and TD children.	Cross-sectional between subjects. Comparison with children with Autism Spectrum Disorder (22N) and TD children (28N).	Behaviour during play opportunities: Behaviour during play related to social entry patterns including disruptive, passive or appropriate styles.	Social Entry Patterns Frequency Scale (Goldstein & Meller, 1999): Parent and teacher-report.	**Strengths:** No significant difference in appropriate or disruptive social entry patterns of children with DLD compared to typically developing peers. **Difficulties:** Significant group difference in passive social entry patterns (*p* = 0.008), with DLD group showing higher passive social entry patterns than TD children.
Marton, Abramoff and Rosenzweig (2005) [48]	19	7–10 years	Mainstream school	To see how social pragmatics relates to social self-esteem in children with DLD.	Cross-sectional between subjects. Comparison with age-matched TD children (19N).	Cooperative behaviour: Negotiation and Conflict resolution skills and related coping strategies. Overall peer competence: Social relations, Conversational skills, Nonverbal communication, Adaptive behaviour	Verbal responses to hypothetical questions relating to negotiation and conflict resolution. Investigators devised their own task. Questionnaires related to social and language competence. Investigators devised their own questions. Parents and teacher-report.	**Strengths:** No significant difference between children with and without DLD on scores of social relations and adaptive behaviour, according to teacher-reports. **Difficulties:** Negotiation and conflict resolution scores significantly lower in children with DLD than TD children (*p* > 0.001). Social competence scores significantly lower in children with DLD than TD children according to parents (*p* > 0.001) and teachers (*p* > 0.01).
Mok, Pickles, Durkin and Conti-Ramsden (2014) [7]	171	7–11 years	Language unit attached to mainstream school (171N), at 7 years.	To see if there are different developmental trajectories for peer relations within children who have a history of DLD.	Longitudinal. Comparison of different subgroups within children with DLD.	Overall peer competence: Peer problems, Prosocial behaviour.	The Rutter Children’s Behaviour Questionnaire (Rutter, 1967): 3 questions relating to peer problems. 7, 8, 11 years. Teacher-report. SDQ (Goodman, 1997): Peer problems, Prosocial subscales. Parent-report. 11 years. Teacher-report.	**Strengths:** 22.2% of children with DLD had no problems/low-level problems with peer relations. 34.5% of children with DLD did not experience peer problems at 8 or 11 years. **Difficulties:** 39.2% of children with DLD developed peer problems during childhood which persisted beyond 11 years.
Pesco (2005) [49]	5	4–5 years	Language unit attached to mainstream school (5N).	To find out how children with DLD use language during peer interactions.	Cross-sectional between subjects. Dyadic interactions with other DLD children and age-matched TD children (6N).	Discourse characteristics: Conversational moves, Principal communicative acts, Subcategory of communicative act during play opportunities. Responses to initiations by play partner. Behaviour during play opportunities: Type of activity engaged in, Interactional context, Verbal exchange.	Observation during four 22–34 min play sessions in class and two 15-min play sessions in playground. Investigators devised their own coding scheme.	**Strengths:** Children with DLD (N = 5) spend more time engaging in interactive play with peers than any other kind of activity (parallel play, solitary play or other) in the playground.* **Difficulties:** Repair acts between DLD-DLD dyads are successful less frequently (45%) than between DLD-TD dyads (67%). *
Redmond (2011) [50]	20	7–8 years	Mainstream school (20N)	To see how behavioural and verbal liabilities contribute to social risk.	Cross-sectional between subjects. Comparison with children with Attention Deficit Hyperactivity Disorder (ADHD, 20N) and same age TD (20N) children.	Overall peer competence: Presence of friendships, Prosocial behaviour. Victimisation: Frequency and type of victimisation.	Child Behavior Checklist (CBCL, Achenbach & Rescorla, 2001): 2 questions relating to presence of friendships. Parent-report. “My Life in School” Questionnaire (MLIS; Sharp, Aurora, Smith, & Whitney, 1994): Verbal bullying index, Physical bullying index, Prosocial index. Self-report.	**Strengths:** For children with DLD (N = 20), narrative skills were significantly positively correlated with prosocial scores (*p* = 0.003). **Difficulties:** Children with DLD had fewer friends than TD children with 1 child having no friends. * Children with DLD (N = 20) reported significantly higher incidences of physical bullying (*p* = 0.04) than TD children. For children with DLD (N = 20), comprehension skills were significantly positively correlated with physical (*p* = 0.019) and verbal (*p* = 0.002) bullying.
Redmond and Rice (2002) [10]	12	6–8 years	Not stated	To assess the stability and reliability of behavioural rating scales in children with DLD.	Longitudinal. Comparison with age-matched TD children (17N) at 6, 7 and 8 years.	Overall peer competence: Withdrawn behaviour, Social problems.	Child Behavior Checklist (CBCL, Achenbach, 1991): Withdrawn index, Social problems index. Parent-report. Teacher Report Form (TRF, Acenback, 1991): Withdrawn index, Social problems index. Teacher-report	**Strengths:** Withdrawn scores significantly decrease between 6 and 8 years (*p* = 0.03, *η²* = 0.39). **Difficulties:** Social problem scores significantly higher for DLD than TD children (*p* = 0.025, *η*² = 0.63). Withdrawn scores significantly higher for DLD than TD children (*p* < 0.001, *η*² = 0.63).
Roth and Clark (1987) [51]	6	5–7 years	Specialist language school (6N)	To characterise the symbolic play and social participation behaviours of children with DLD.	Cross-sectional between subjects. DLD-DLD dyads compared to language matched (younger) TD-TD dyads (8N).	Behaviour during play opportunities: Type of participation in play, Type of symbolic play, Developmental level of symbolic play.	Observation during two 15-min and one 45-min play sessions. Behaviour coded using the Scale of Social Participation in Play (SSPP, Tizard, Philps & Plewis, 1976), the Symbolic Play Test (SPT, Lowe & Costello, 1976) and Brown et al’s (1975) modification of Lunzer’s (1959) Scale of Organization of Behavior for Use in the Study of Play.	**Strengths:** No relevant strengths. **Difficulties:** Pretend play scores significantly lower for DLD than language-matched TD children (*p* < 0.05). Frequency of non-play behaviours significantly higher for DLD than language-matched TD children (*p* < 0.05). Frequency of solitary-play behaviours significantly higher for DLD than language-matched TD children (*p* < 0.05). Frequency of parallel play behaviours significantly lower for DLD than language-matched TD children (*p* < 0.05).
Weitzner (1981) [52]	4	4 years	Not stated	To characterise the manner in which children with DLD use requests during interactions.	Cross-sectional within subjects. Dyadic interactions between children with DLD and same age TD peer (1N) and adult (1N).	Discourse characteristics: Type of request, Interactive context surrounding requests, Characteristic form of requests. Behaviour during play opportunities: Non-verbal behaviour used to make requests.	Observation during play sessions, length of time not specified. Investigators devised their own coding scheme.	**Strengths:** Children with DLD were able to make verbal requests (N = 4). * Requests were used in different ways including to introduce new topics, for affirmation and for clarification. * Difficulties: Some children used non-verbal requests more frequently than verbal requests (N = 2). *

TD = typically developing. * Parametric tests of significance not reported.

**Table 3:** Table showing proportion of studies containing samples enrolled in different types of school.

School Type	Total Proportion of Studies
Mainstream only	39.3%
Specialist schools or language units or specialist classes attached to mainstream schools only	21.4%
Mixed sample (some mainstream, some specialist schools/language units)	7.1%
Educational provision not stated	32.1%

**Table 4:** Table to show the total number of studies measuring different variables related to peer interaction skills within identified skill areas. Each study could include multiple variables within multiple skill areas.

Skill Area and Total Studies per Skill Area	Variables Measured	Total Studies per Variable N (%)
Overall peer competence	Prosocial behaviour	9 (32.1%)
(*N* = 13, 46.4%)	Peer problems	7 (25.0%)
	Level of social withdrawal	4 (14.3%)
	Ability to form reciprocal friendships	3 (10.7%)
	Social competence with peers	2 (7.1%)
	Sociometric ratings of peer liking	1 (3.6%)
	Level of assertiveness	1 (3.6%)
Behaviour during play opportunities	Type of interaction/play behaviour	5 (17.9%)
(*N* = 11, 39.3%)	Access behaviours	3 (10.7%)
	Sophistication of social pretend play	2 (7.1%)
	Social interactions directed to peers	2 (7.1%)
	Non-verbal behaviour during play	1 (3.6%)
Discourse characteristics	Type of content in discourse	5 (17.9%)
(*N* = 9, 32.1%)	Turn-taking	3 (10.7%)
	Topic maintenance/manipulation	2 (7.1%)
	Amount of talk	2 (7.1%)
	Formation of requests	2 (7.1%)
Cooperative behaviour	Conflict resolution knowledge	3 (10.7%)
(*N* = 6, 21.4%)	Joint decision making on cooperative task	2 (7.1%)
	Validating comments during cooperative tasks	1 (3.6%)
Victimisation	Frequency of victimisation	3 (10.7%)
(*N* = 4, 14.3%)	Type of victimisation	2 (7.1%)

## Appendix A

**Table A1:** Table to show the searches conducted in the current systematic review.

Search Number	Search Terms
1	(interact OR interaction OR interactions OR engage OR engagement OR communicate OR communication OR talk OR talking OR verbal OR “non-verbal”) AND (child OR children OR childhood OR friend OR friends OR peer OR peers OR classmate OR classmates OR preschool OR “pre-school” OR preschoolers OR “pre-schoolers” OR kindergarten OR kindergartners) AND (“language disorder” OR “language disorders” OR “language problems” OR “impaired language” OR “language impairment” OR “language impaired” OR “developmental aphasia” OR “developmental dysphasia” OR “language deficit” OR “language learning impairment” OR “language delay”)
2	(play OR playing OR playground OR play-ground OR social OR socialise OR socially OR sociability OR unsociability OR “non-social” OR prosocial OR behaviour OR behavior OR behavioural OR behavioral) AND (child OR children OR childhood OR friend OR friends OR peer OR peers OR classmate OR classmates OR kindergarten OR kindergartners OR preschool OR “pre-school” OR preschoolers OR “pre-schoolers”) AND (“language disorder” OR “language disorders” OR “language problems” OR “impaired language” OR “language impairment” OR “language impaired” OR “developmental aphasia” OR “developmental dysphasia” OR “language deficit” OR “language learning impairment” OR “language delay”)
3	(“socio-emotional” OR “socio emotional” OR negotiate OR negotiation OR withdraw OR withdrawal OR withdrawn OR shy OR shyness OR reticence OR reticent OR conflict OR aggression OR aggressive) AND (child OR children OR childhood OR friend OR friends OR peer OR peers OR classmate OR classmates OR kindergarten OR kindergartners OR preschool OR “pre-school” OR preschoolers OR “pre-schoolers”) AND (“language disorder” OR “language disorders” OR “language problems” OR “impaired language” OR “language impairment” OR “language impaired” OR “developmental aphasia” OR “developmental dysphasia” OR “language deficit” OR “language learning impairment” OR “language delay”)

## Appendix B

**Table A2:** Table to show the scoring guidelines used for the quality appraisal, based on criteria by Alderfer et al. (2010) [25].

		Score Assigned		
Quality Criteria	Type of Study	1	2	3
Explicit scientific context and purpose	Qualitative, Quantitative	Poorly done	A rationale but not all clear	All: Clear rationale for study, theoretically and/or empirically based questions/predictions, specified purpose
Methods	Qualitative, Quantitative	Poorly done	Appropriate design and analysis but not enough description for replication	Appropriate design and analysis for question posed, enough description of methods to allow for replication
Measurement reliability	Quantitative	Poorly done	Adequate statistical methods but not always best and interpretation adequate but not always appropriate	Reliable measurement of variables, adequate statistical methods used, appropriate interpretation of results
Statistical power	Quantitative	Not sufficient	Adequate but could be improved.	Statistical power is sufficient
Internal validity	Quantitative	Poorly done	Groups are comparable on most aspects but adequate steps not always taken. Reverse relationship is possible or 3rd variable is possible, but explained in discussion.	Groups are comparable on all aspects aside from IV, and if not adequate steps are taken. Only passage of time occurs between assessments if measured over time. Reverse relationship not possible in correlation design, and not explainable by 3rd variable
Valid measures	Quantitative	Poorly done	Variables are adequately operationalised but some limitations	Variables are appropriately operationalised and measured (results would be the same if other measures were used)
External validity	Quantitative	No evidence that findings can be generalised beyond the study setting	The findings can be generalised but there are some limitations	The findings are generalizable to the target population, real world, and across time periods
Examples	Qualitative	No examples given	Some examples but not always well explained	Examples illustrate conclusions, help reader understand analytic procedure and form possible alternative meanings of the data
Findings framework	Qualitative	No attempt to integrate findings into a framework	Some attempt to integrate findings into a framework but not explained clearly	Findings are integrated into a framework
Author perspective	Qualitative	No attempt to specify theoretical orientation	Authors make some attempt to orient their perspective but not explained fully and/or clearly	Authors specify their theoretical orientation and expectations that might impact the interpretation of data
Reader perspective	Qualitative	Poorly done	Accurate perspective of topic area but not always clear/Understandable account but not always accurate representation of topic area	Accurate, understandable perspective of topic area
Appropriate range	Qualitative	Poorly done	Data is based on more than one situation and has been studied fairly systematically but not there is room for improvement	Data is based on a suitable range of informants and situations and/or the topic has been studied systematically and comprehensively within the specified population or situation
Credibility checks	Qualitative	Poorly done	Some attempt to verify findings but some limitations	Verification of findings with participants, across multiple coders, or through methodological triangulation
Situated sample	Qualitative	No attempt to situate the sample	A weak attempt to situate the sample	Sample is described such that the reader can judge for whom the findings are relevant
Appropriate discussion	Qualitative, Quantitative	Poorly done	Appropriate discussion and largely appropriate conclusion but limitations not fully discussed	Appropriate discussion with limitations notes and conclusion appropriate to data gathered
Contribution to knowledge	Qualitative, Quantitative	Barely	Adequate attempt	Strong attempt. Contributing something new or validating past results

## Appendix C

**Table A3:** Table to Show Quality Appraisal Scores Assigned to Studies Included in Review.

Item	Score
Bakopoulou and Dockrell (2016) [28]	2.89
Brinton, Fujiki, Montague and Hanton (2000) [29]	2.22
Campbell and Skarakis-Doyle (2011) [30]	2.56
Conti-Ramsden and Botting (2004) [31]	3.00
Craig and Gallagher (1986) [32]	2.22
Craig and Washington (1993) [33]	2.31
DeKroon, Kyte and Johnson (2002) [34]	2.25
Edmonds and Haynes (1988) [35]	2.56
Farmer (2000) [36]	2.78
Fey, Leonard and Wilcox (1981) [37]	1.94
Fujiki et al. (2013) [38]	2.11
Fujiki and Brinton (1991) [39]	2.22
Fujiki, Brinton, Isaacson and Summers (2001) [11]	2.89
Gibson, Hussain, Holsgrove, Adams and Green (2011) [40]	2.78
Grove, Conti-Ramsden and Donlan (1993) [41]	2.56
Guralnick, Gottman and Hammond (1996) [42]	2.89
Lederer (1996) [43]	2.22
Levickis et al. (2017) [44]	2.89
Lindsay and Dockrell (2012) [45]	2.78
Liiva and Cleave (2005) [46]	2.67
Margolis (2001) [47]	2.44
Marton, Abramoff and Rosenzweig (2005) [48]	2.50
Mok, Pickles, Durkin and Conti-Ramsden (2014) [7]	2.44
Pesco (2005) [49]	2.50
Redmond (2011) [50]	2.56
Redmond and Rice (2002) [10]	2.33
Roth and Clark (1987) [51]	2.44
Weitzner (1981) [52]	1.33
